# Non-invasive surveillance for *Plasmodium* in reservoir macaque species

**DOI:** 10.1186/s12936-015-0857-2

**Published:** 2015-10-12

**Authors:** Josephine E. Siregar, Christina L. Faust, Lydia S. Murdiyarso, Lis Rosmanah, Uus Saepuloh, Andrew P. Dobson, Diah Iskandriati

**Affiliations:** Eijkman Institute for Molecular Biology, Jakarta, Indonesia; Department of Ecology and Evolutionary Biology, Princeton University, Princeton, NJ 08544 USA; Pusat Studi Satwa Primata, Institut Pertanian Bogor, Bogor, Indonesia

**Keywords:** Malaria, *Macaca fascicularis*, *Macaca nemestrina*, Non-invasive sampling, Zoonotic surveillance

## Abstract

**Background:**

Primates are important reservoirs for human diseases, but their infection status and disease dynamics are difficult to track in the wild. Within the last decade, a macaque malaria, *Plasmodium knowlesi*, has caused disease in hundreds of humans in Southeast Asia. In order to track cases and understand zoonotic risk, it is imperative to be able to quantify infection status in reservoir macaque species. In this study, protocols for the collection of non-invasive samples and isolation of malaria parasites from naturally infected macaques are optimized.

**Methods:**

Paired faecal and blood samples from 60 *Macaca fascicularis* and four *Macaca nemestrina* were collected. All animals came from Sumatra or Java and were housed in semi-captive breeding colonies around West Java. DNA was extracted from samples using a modified protocol. Nested polymerase chain reactions (PCR) were run to detect *Plasmodium* using primers targeting mitochondrial DNA. Sensitivity of screening faecal samples for *Plasmodium* was compared to other studies using Kruskal Wallis tests and logistic regression models.

**Results:**

The best primer set was 96.7 % (95 % confidence intervals (CI): 83.3–99.4 %) sensitive for detecting *Plasmodium * in faecal samples of naturally infected macaques (n = 30). This is the first study to produce definitive estimates of* Plasmodium* sensitivity and specificity in faecal samples from naturally infected hosts. The sensitivity was significantly higher than some other studies involving wild primates.

**Conclusions:**

Faecal samples can be used for detection of malaria infection in field surveys of macaques, even when there are no parasites visible in thin blood smears. Repeating samples from individuals will improve inferences of the epidemiology of malaria in wild primates.

**Electronic supplementary material:**

The online version of this article (doi:10.1186/s12936-015-0857-2) contains supplementary material, which is available to authorized users.

## Background

Non-human primates (NHPs) are important hosts of zoonotic diseases: they can share pathogens with humans and act as reservoirs for several emerging infectious diseases of pandemic proportions [1–4]. NHPs are also susceptible to human pathogens that can have mild to catastrophic impacts on populations [5–9]. In order to predict zoonotic risk and understand conservation implications of pathogen exchange between humans and NHPs, it is essential to understand infectious disease dynamics within wild primate populations.

**Fig. 1:**
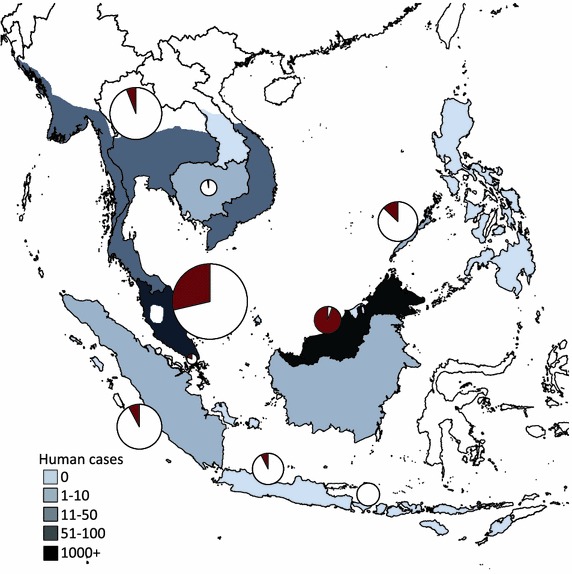
Macaque malaria in long-tailed macaques and humans in Southeast Asia. The number of human *P. knowlesi* cases that have originated from the location is represented in shades of grey. The maximum range of *Macaca fascicularis* [50] is greater than the extent of known *P. knowlesi* spillover cases. *Pie* charts depict cumulative results of parasitological and molecular surveys of malaria (all five *Plasmodium* species) in long-tailed macaques, with the size of the circle corresponding to sample size from the given location. Referenced raw data are given in Additional file 1

Primates are infected with at least thirty *Plasmodium* parasites globally [10, 11]. Spillover of NHP malaria has been suspected in cases in the Amazon [12–14] and a tourist returning from Central Africa [15]. On a much larger scale, a monkey malaria, *Plasmodium knowlesi*, has emerged in human populations across Southeast Asia (Fig. 1, Additional file 1). The parasite species has been recorded, and is presumably endemic, in wild populations of two macaque species (*Macaca fascicularis* and *Macaca nemestrina*) and two leaf monkeys (*Presbytis femoralis* and *Trachypithecus obscurus*) [16–19]. These primates can be co-infected with up to five species of *Plasmodium* parasite [20], but most morphological surveys report moderate (10–30 %) malaria prevalence in long-tailed macaques (Fig. 1, see Additional file 1). While spillover cases have increased in incidence in some regions over the last decade [21, 22], there is no evidence for human-to-vector-to-human transmission, therefore quantifying malaria dynamics within reservoir populations is essential.

While large foci of zoonotic malaria is currently restricted to Southeast Asia, this spillover presents a unique opportunity to document processes underlying the evolution of malaria. *Plasmodium falciparum* and *Plasmodium vivax*, the most important human malaria parasites, are the result of host switches from NHPs [15, 23–26]. Malaria is the most important human infectious disease globally, causing hundreds of thousands of deaths and disease in millions annually [27]. Understanding the dynamics in wild primates will only improve our understanding of the underlying drivers of infection in humans.

Non-invasive sampling methods are the primary tool for primate infectious disease ecology, because many times logistical challenges and ethical considerations prohibit collection of blood and tissues. Non-invasive sampling methods have been used to successfully study parasites across taxa, with the majority of studies focusing on parasites that are transmitted through a faecal-oral route [28–30]. However, researchers have recently used faecal samples to study malaria parasites in apes in Central Africa and have been able to isolate *Plasmodium* DNA from human stool samples [15, 23, 25, 26, 31] (see Additional file 2). While isolation of *Plasmodium* DNA has been successful, there are no robust estimates of how presence of *Plasmodium *DNA in faeces translates to disease status and prevalence among populations.

The objectives of this study were to determine if non-invasive sampling is a reliable method for tracking *Plasmodium* reservoir dynamics in monkeys of Southeast Asia. Paired samples from semi-captive Indonesian long-tailed macaques (*M. fascicularis*) and pig-tailed macaques (*M. nemestrina*) that were naturally infected were used for validation. Protocols were developed and optimized to detect any of five *Plasmodium* species that infect these macaques.

## Methods

### Sample collection

Individual macaques of *M. facscicularis* originating from Lampung (n = 30) and Palembang (n = 30), Sumatra, and *M. nemestrina* originating from Java (n = 4) were used as sources for paired blood and faecal samples. Animals were sedated for blood collection as a part of routine health screening at the breeding facilities in West Java. Blood samples were collected in EDTA tubes and, at the same time, thin blood smears were made from all *M. fascicularis*. Faecal swabs were taken at the time of sedation for the *M. fascicularis* (200–300 mg) and whole faeces were obtained from cages of *Macaca nemestrina.* Initial pilot work with *M. nemestrina* samples (see Additional file 3) demonstrated that samples stored in RNAlater ^®^ (Qiagen) had consistently higher DNA yield, so all faecal samples were stored in sterile vials in a 1:1 ratio with RNAlater. Samples were transferred to −20 °C as soon as possible (within 24 h) until extraction. Paired blood was frozen at −20 °C immediately after collection. All procedures involving animals were approved by Institutional Animal Care and Use Committee, Primate Research Center, Bogor Agricultural University number IPB PRC-13-A012.

### Molecular analysis

DNA was extracted from faecal samples using a modified protocol from the QIAamp Mini Stool Kit (Qiagen^®^). Samples were lysed in ASL buffer overnight with intermident vortexing to increase DNA yield; the rest of the protocol was followed as per manufacturer instructions. Filter pipette tips were used in all stages of processing to prevent cross-contamination. Screening for malaria parasites was done using three primer sets targeting cytochrome b (cytb) and one set targeting a fragment of the mitochondrial small subunit rRNA (ssrRNA) in *Plasmodium* (Table 1). Each  cytb primer set was tested in two replicates for each faecal and blood sample originating from one individual. The ssrRNA primers were only tested on a subset (n = 32) due to limited reagents.

**Table 1:** Primers and sensitivity for amplifying *Plasmodium* from faecal samples

ID	Primer sets	Nest?^a^	Sequence	Product length (bp)	Gene	Reference for primers
ssrRNA	PfF4595 PfR5019	reamp	GATTACAGCTCCCAAGCAAAC GTTTAGCCAGGAAGTCAGCGTC	424	ssrRNA	This paper
cytb1	PfF3700 PfR4615	nest1a	TGGATGGTGTTTTAGATACATGC GTTTGCTTGGGAGCTGTAATC	915	cytb	This paper
cytb1	PfF3700 PfR4102	nest2a	TGGATGGTGTTTTAGATACATGC GCTGTATCATACCCTAAAG	402	cytb	This paper
cytb2	PfF3368 PfR4102	nest1b	TGCCTAGACGTATTCCTGAT GCTGTATCATACCCTAAAG	734	cytb	This paper
cytb2	PfF3368 PfR3717	nest2b	TGCCTAGACGTATTCCTGAT TATCTAAAACACCATCCACTCCA	349	cytb	This paper
cytb3	qPlasm1f qPlasm1r	nest1c	CTGACTTCCTGGCTAAACTTCC CATGTGATCTAATTACAGAAYAGGA	170	cytb	[32]
cytb3	qPlasm2f qPlasm2r	nest2c	AGAAAACCGTCTATATTCATGTTTG ATAGACCGAACCTTGGACTC	90	cytb	[32]

Primers are all listed in the 5′ to 3′ end

^a^Nested reactions are indicated: the first primer set is denoted with ‘1’ and the second set is denoted with ‘2’, and nested primer sets can be differentiated by the letter following the number. Sensitivity is only given for the complete nested reaction. The primer set targeting the ssrRNA segment was used twice in a re-amplification reaction to increase yield

Three sets of nested PCR were used to amplify the cytochrome *b* gene; two sets were designed specifically for this study. The first nested primer set was PfF3700/PfR4615 and targeted a 915 bp fragment. The second PCR reaction utilized the first product with primer set PfF 3700/PfR4102 targetting a 402 bp fragment. The first nested PCR reaction was run with 2 μL of template, while the second was run with 1 μL of product from the first round reaction. Both PCRs were run using KAPA Taq DNA Polymerase (KAPA Biosystems, USA) under the following conditions: 5 min denaturation at 94 °C, 30 cycles of 15 s denaturation at 94 °C, 15 s annealing at 50 °C, 45 s extension at 72 °C, and a final extension of 5 min at 72 °C.

The second nested reaction designed for this study first used the primer set PfF3368/PfR4102 targeting  a 734 bp fragment, followed by a nested reaction with the primer set PfF3368/PfR3717 targetting a 349 bp fragment. The reaction conditions were the same as above.

PCR amplifying a fragment of ssrRNA of mitochondrial *Plasmodium* utilized reamplification with primer set PfR4595/PfR5019. The amplified target was a 424 bp fragment (Table 1). Both PCRs were run using KAPA Taq DNA Polymerase (KAPA Biosystems, USA) under the following conditions: 5 min denaturation at 94 °C, 30 cycles of 15 s denaturation at 94 °C, 15 s annealing at 56 °C, 45 s extension at 72 °C, and a final extension of 5 min at 72 °C.

An additional nested PCR was conducted with primers developed for detection of *Plasmodium* in chimpanzees [32]. The first round of PCR was run with primers qPlasm1f/qPlasm1r targeting a 170 bp segment of cytb under the following conditions: 3 min denaturation at 96 °C, 30 cycles of 30 s denaturation at 96 °C, 30 s annealing at 52 °C, 1 min extension at 72 °C, and a final extension of 5 min at 72 °C. The second round of PCR was conducted with primer qPlasm2f/qPlasm2r resulting in a 90 bp product under the following conditions: 5 min denaturation at 95 °C, 30 cycles of 15 s denaturation at 96 °C, 15 s annealing at 50 °C, 30 s extension at 60 °C, and a final extension of 5 min at 60 °C.

All PCR reactions were run  with a 25 μl PCR mixture: 1–2 μl of template, 2.5 μl of 10X Kapa Taq PCR buffer (standard Tris-ammonium sulfate-based buffers containing 15 mM MgCl_2_; KapaBiosystems, USA), 10 pmol of each primer, 100 μM of dNTPs (Promega), 0.5 U of *Taq* DNA Polymerase (KapaBiosystems, USA). All PCR reactions were run with negative and positive (*P. inui* isolate) controls.

Positive PCR products were purified (Qiagen PCR Purification Kit) and directly sequenced using Forward and Reverse primer for PCR cytochrome *b* and fragment ssrRNA of mitochondrial *Plasmodium* in an ABI 3130 XL Genetic Analyzer. The sequences obtained were BLAST to the reference sequences and aligned using the BioEdit program (Ibis Biosciences, CA, USA) to determine identity of *Plasmodium* species.

### Microscopy

Thin blood smears were prepared by spreading two drops of fresh blood on a slide. Slides were dried and fixed with methanol. Dried slides were stained with 10 % Giemsa solution. Each slide was examined under light microscope in all fields of slide by at least two trained malariologists. Parasite morphology was used to identify species following Coatney et al. [11].

### Statistical analysis

Several statistics were calculated for each primer set used to detect *Plasmodium* species in faecal samples. (1) Sensitivity was calculated as the proportion of infected macaques (as defined by PCR positive in blood) that were PCR positive by faecal screening. Because sample sizes were low, 95 % confidence intervals were calculated using a Wilson score interval [33]. (2) Specificity was measured as the proportion of uninfected macaques (again, defined as PCR negative in the blood) identified as PCR negative in the faecal samples. The last statistic in the diagnositics was the (3) false negative rate (FNR), which was the number of false negatives (PCR negative in faeces but PCR positive in blood) divided by the sum of the false negatives and true positives. A lower false negative rate is preferred for estimating population prevalence.

**Fig. 2:**
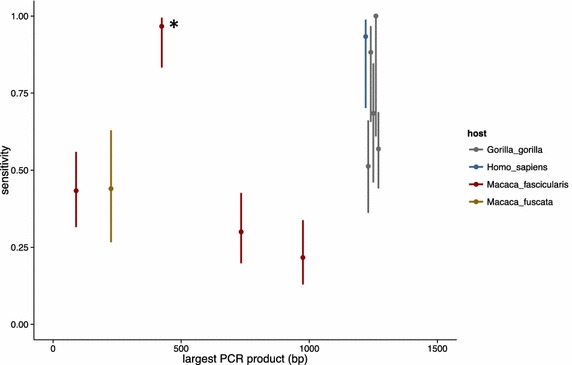
Sensitivity of faecal screening for detection of *Plasmodium* infections in primates. Estimates for the sensitivity of screening faecal samples for *Plasmodium* from this study and previously published studies are shown (Additional file 2, [26, 31, 40]). The largest PCR product from all samples of gorillas and humans was 1230 bp, but are shown staggered in the figure for visual clarity. Confidence intervals are calculated using the Wilson interval for samples under 40 and the Agresti-Coull method for datasets that have 40 or greater sampling units. All calculations were done using the *binom* package [51] in R v. 3.0.2 [34]. The *asterisk* indicates the sensitivity of the best primers from this study

Sensitivity of the screening in this study was compared to other studies using primate faecal samples (including humans) to detect malarial DNA (Fig. 2, raw data, Additional file 2). A logisitic regression was fit to data from all the studies, using amplicon length, average adult female mass of host species, storage media, and PCR protocols as predictor variables in a logisitc regression using function *glm* in R vs 3.0.2 [34]. Predictor variables were chosen because they had data for all the studies in the analysis and were expected to influence DNA quality and diagnostic ability. If any of the variables significantly influence the sensitivity of protocols running a logisitic regression should be able to determine its magnitude and directionality.

## Results

### Sensitivity of primers for screening macaque faeces

Overall, 34 of 64 macaques (53.1 %) were infected with malaria (Table 2). Active infections were determined with PCR of blood samples  and was considered a reflection of the true prevalence of infection. Thin blood films detected 17 (56.7 %) of the 30 positive *Macaca fascicularis* individuals.

**Table 2:** Malaria infections of *Macaca* individuals determined by invasive samples

Species	Origin	N	PCR + blood	Sensitivity of microscopy
*M. nemestrina*	West Java	4	4 (100 %)	NA
*M. fascicularis*	Lampung	30	6 (20 %)	3 (50 %)
*M. fascicularis*	Palembang	30	24 (80 %)	14 (58.3 %)

Primer sets varied in their sensitivity and false negative rate for detection of *Plasmodium* in faecal samples, but not their specificity (Table 3). Faecal screening never yielded a false positive. The maximum sensitivity was achieved with ssrRNA primers and was 96.7 % (83.3–99.4 %).

**Table 3:** Sensitivity, specificity, and false negative rate of each primer set in faecal samples

	Number of faecal samples	PCR + in paired blood sample	1 PCR run			2 PCR runs		
			Sensitivity^a^	Specificity	FNR^b^	Sensitivity	Specificity	FNR
ssrRNA	32	30	96.7 % (83.3–99.4 %)	100 %	0.03	NA	NA	NA
cytb-a	64	34	29.4 % (16.8–46.2 %)	100 %	0.71	29.4 % (19.9–41.2 %)	100 %	0.71
cytb-b	64	34	29.4 % (16.8–46.2 %)	100 %	0.71	23.5 % (14.9–35.0 %)	100 %	0.76
cytb-c	64	34	41.2 % (26.4–57.8 %)	100 %	0.59	41.2 % (30.2–53.0 %)	100 %	0.59

^a^The sensitivity is the percentage of infected macaques that are positive by PCR from faecal samples. 95% confidence intervals (CI) are given in parenthesis

^b^FNR is the false negative rate, or the number of false negatives divided by the sum of false negatives and true positives

Because of the limited volume of samples, DNA extraction could not be repeated. It is possible that extraction steps affected the estimates of sensitivity in these samples. Additional studies are warranted to examine if repeating extractions, in addition to PCR reactions, can increase the sensitivity of the assay.

### Species of *Plasmodium*

*Plasmodium* cytochrome *b* genes from all positive blood samples of *M. fascicularis* were sequenced. Sequencing ssrRNA was insufficient to determine species identity. The majority of malaria infections were caused by *Plasmodium inui* (23 samples from Palembang, six samples from Lampung). Two isolates (one from each Palembang and Lampung) clustered more closely with GenBank samples identified as *Plasmodium fieldi* and *Plasmodium cynomolgi* but a specific species could not be assigned.

### Sensitivity of PCR for *Plasmodium* across primate species

A Kruskal–Wallis test on the entire dataset failed to demonstrate a significant difference in sensitivity across all samples (df = 10, p = 0.4405). Although this shows that differences were not significantly different globally, multivariate regressions were used to determine if predictor variables could explain differences in sensitivity. Both logistic regression and quasibinomial regressions were insufficient in elucidating factors that affected variation in the sensitivity. None of the predictor variables significantly impacted sensitivity, suggesting the models are not including all relevant variables. Increasing sample sizes and consistent molecular methods across a diversity of species will help elucidate important predictors of sensitivity of faecal samples for *Plasmodium* detection.

## Discussion

Active macaque malaria infections can be detected in faecal samples using protocols developed in this study with 96.7 % (95 % CI: 83.3–99.4 %) sensitivity. This assay is significantly better than thin blood smears and presents an alternative, non-invasive method to measure pathogen prevalence in wild populations.

To optimize detection of low concentrations of parasite DNA, short amplicons in of DNA were targeted. *Plasmodium* species have a small mitochondrial genome (mtDNA), spanning only 6 kb but with copy numbers ranging from 20 to 100 per parasite [35, 36], whereas other common molecular targets (i.e., 18S rRNA) are present in low copy numbers [37]. Several primers that amplified segments greater than 700 bp were unsuccessful in amplifying parasite DNA. The primer set with the highest sensitivity targetted the mitochondrial ssrRNA. Although primers detecting ssrRNA had higher sensitivity, species identity had to be determined with cytb isolates. The ssrRNA region of *Plasmodium* has been shown to be inaccurate at identifying *Plasmodium* species [38]. The amplicon length of cytb also affected the ability to determine species—identification became impossible with shorter amplicons. For diagnostics of malaria infection, the results of this study show that a primary screening with ssrRNA to detect infections is most accurate. Positive samples can then be identified with cytb amplicons.

Malaria infections are notoriously hard to detect in macaques by microscopy. Splenectomy was a common procedure historically to increase parasitaemia for detection of parasites and for work in the laboratory [39]. Chronic infections lasting for years are believed to be characteristic of several species of macaque *Plasmodium* [11]. This study demonstrates detection in naturally infected populations and shows that  even low level infections can be detected.

The protocols outlined here will enable a more critical examination of the epidemiology of malaria in wild primate populations. Primate malaria research has relied on observations of mostly adult animals and, analogous to humans, the burden of malaria may fall on young animals. In fact, a recently published paper demonstrates anaemia and high parasitaemia associated with *Plasmodium reichnowi* in a juvenille chimpanzee [40]. A distinct age-class-prevalence curve has been recorded in howler monkeys, *Alouatta* species, from Panama [41] and chimpanzees, *Pan troglodytes,* in Cameroon [42]. More work in wild primate populations will inform the natural course of infections across the diversity of parasites. Epidemiology will most likely reflect a combination of host susceptibility (age, immune status, reproductive status, etc.), pathogen characteristics (periodicity, peak parasitaemia) and local environment affecting the epidemiology.

Using non-invasive faecal samples poses several challenges for evaluating the epidemiology of primate malaria. A direct comparison of faecal *Plasmodium* DNA and blood parasitaemia is not possible, and variation between timepoints within an individual is expected to be much greater than observed in the blood [41]. In laboratory-infected mice, *Plasmodium* DNA can be isolated during the pre-erythocytic phase, suggesting that presence in faecal samples is not indicative of an active infection [41]. Experimental infections of Japanese macaques [40] also demonstrate that *Plasmodium* DNA is detectable in faeces for days after an animal is treated and peripheral blood is negative. Although this study did not support previous findings, researchers are cautioned from drawing conclusions about the epidemiology of malaria from *Plasmodium* isolated from faecal samples.

In contrast to a study of an experimentally *P. knowlesi*-infected *Macaca fuscata* [43], parasite DNA was never found in faeces of uninfected macaques. In the experimental host, *P. knowlesi* DNA was detected in faecal samples for 12 days after the parasites were no longer present in blood samples. Abkallo and colleagues [44] concluded that parasite clearance caused accumulation of *Plasmodium* DNA in the gall bladder that was slowly excreted into faeces via bile. However, there were no false positives in faecal samples of naturally infected macaques in this study. This may reflect the chronicity of infections in these natural hosts and suggests that detection in faeces can be used to infer active infection in naturally infected macaques.

Other sources of non-invasive samples may have higher sensitivity, but there is a trade-off for ease of collection. In humans, sensitivity of PCR in saliva samples can reach 85 % [45, 46], with an ELISA study reporting 100 % sensitivity (although low sample size, n = 8) [47]. Higher parasitaemias increase probability in detecting parasites [48], so these methods may not be as robust with chronic infections. Protocols for sampling saliva in wild macque populations have been developed [49], and could be used in malaria-endemic populations for higher sensitivity in non-invasive screening.

## Conclusions

Detection of *Plasmodium* DNA from faecal samples is complicated by degradation of DNA, absence of a clear relationship between parasitaemia and presence in faecal samples, and chronic infections in natural populations. These challenges require using robust extraction methods and amplifying short segments of DNA. This study demonstrates that amplification of parasite DNA is repeatable, even with low parasitaemia. Sensitivity of faecal screening is significantly higher than thin blood smears and is more tractable for field surveys. *Plasmodium* DNA can be amplified from faecal samples of naturally infected macaques. Repeated samples from individuals improves the ability to detect malaria infections and could improve understanding of malaria epidemiology in wild primates.

## Additional files

Additional file 1: Raw data of long-tailed macaque (*Macaca fascicularis*) malarias. Includes surveys of *Plasmodium coatneyi, P. cynomolgi, P. fieldi, P. knowlesi,* and *P. inui* in natural hosts and humans across Southeast Asia. Additional file 2: Non-invasive surveys for primate malarias. Raw data from studies that quantified *Plasmodium *detection from non-invasive samples. Additional file 3: Optimal storage techniques. Data on DNA yield from faecal samples stored with four different protocols that varied in temperature and media.
