# Supplementary material for: Non-invasive surveillance for Plasmodium in reservoir macaque species
Source: Malar J. 2015 Oct 12;14:404. doi: 10.1186/s12936-015-0857-2 (PMC4603874; doi:10.1186/s12936-015-0857-2)
Supplement: Additional file 2: — Non-invasive surveys for primate malarias. Raw data from studies that quantified Plasmodium detection from non-invasive samples. [file 12936_2015_857_MOESM2_ESM.docx]

### Additional file 2. Sensitivity of non-invasive fecal samples for malaria in primates (raw data)

We conducted a literature search for studies that examined the sensitivity of noninvasive samples from primates. Search terms included: non-invasive, faecal, fecal, ‘AND’ malaria or *Plasmodium*. Studies that were included were only those that had a way to measure sensitivity- this ranged from paired blood samples to repeated samples from the same individual on the same day.

| Study | Species | Mass (kg)^◆^ | Infection | non-invasive  sample | comparison | method* | primers^+^ | storage media | amplicon  length | first nest | gene | location | X | N | Sensitivity  (95% CI)^ |
| --- | --- | --- | --- | --- | --- | --- | --- | --- | --- | --- | --- | --- | --- | --- | --- |
| Kawai *et al* [1] | Japanese macaque (*Macaca fuscata)* | 10.11 | lab | fecal, urine | paired blood samples | nPCR | A | RNAlater | 131bp | 226bp | cytb |  | 11 | 25 | 0.59 (0.36-0.82) |
| Liu *et al*  [2] | Gorillas (*Gorilla gorilla*) | 112.58 | natural | fecal | multiple samples from one individual | nPCR | B | RNAlater | 958bp | 1230bp | cytb | Cameroon  Republic Congo  DR Congo  Uganda  Central Afr Repub  total | 20  13  33  15  6  87 | 39  19  58  17  6  139 | 0.51 (0.36-0.66)  0.68 (0.47-0.87)  0.57 (0.44-0.69)  0.86 (0.71-0.99)  0.93 (0.74-1.00)  0.63 (0.54-0.70) |
| Jirku *et al*  [3] | humans | 58.54 | natural | fecal | paired blood samples | nPCR | B | 95% EtOH | 928bp | 1230bp | cytb |  | 14 | 15 | 0.91 (0.77-1.00) |
| Abakallo  *et al* [4] | macaque (no species) | 6 (est) | natural | fecal | paired blood samples | nPCR | C | RNAlater | 750bp | 1230bp | cytb |  | 1 | 1 | 0.75 (0.23-1.00) |

^◆^Adult body mass was obtained from the PanTHERIA database on life history characteristics [5]

*nPCR= nested PCR

^+^primer sets= A (Forward primers from Putaporntip et al. [6] and reverse primers from Tanizaki et al. [7]); B(DW2-F + DW4-R ; CYTB1-F + CYTB2-R [8]); C(DW2-F + DW4-R; NCYBINF + NCYBINR [4])

^ When sample sizes(N) <40 we used the Wilson interval for calculating the binomial proportion confidence intervals, whereas when N> 40 we used Agresti-Coull for calculated confidence intervals

**References**

1. Kawai S, Sato M, Kato-Hayashi N, Kishi H, Huffman MA, Maeno Y, Culleton R, Nakazawa S: **Detection of Plasmodium knowlesi DNA in the urine and faeces of a Japanese macaque (Macaca fuscata) over the course of an experimentally induced infection.** *Malaria Journal* 2014, **13**.

2. Liu W, Li Y, Shaw KS, Learn GH, Plenderleith LJ, Malenke JA, Sundararaman SA, Ramirez MA, Crystal PA, Smith AG, et al: **African origin of the malaria parasite Plasmodium vivax.** *Nature Communications* 2014, **5:**1-10.

3. Jirků M, Pomajbíková K, Petrželková KJ, Hůzová Z, Modrý D, Lukeš J: **Detection of Plasmodiumspp. in Human Feces.** *Emerging Infectious Diseases* 2012, **18**.

4. Abkallo HM, Liu W, Hokama S, Ferreira PE, Nakazawa S, Maeno Y, Quang NT, Kobayashi N, Kaneko O, Huffman MA, et al: **DNA from pre-erythrocytic stage malaria parasites is detectable by PCR in the faeces and blood of hosts.** *INTERNATIONAL JOURNAL FOR PARASITOLOGY* 2014, **44:**467-473.

5. Jones KE, Bielby J, Cardillo M, Fritz SA, O'Dell J, Orme CDL, Safi K, Sechrest W, Boakes EH, Carbone C, et al: **PanTHERIA: A Species-Level Database of Life History, Ecology, and Geography of Extant and Recently Extinct Mammals.** *Ecology* 2009, **90:**2648-2648.

6. Putaporntip C, Buppan P, Jongwutiwes S: **Improved performance with saliva and urine as alternative DNA sources for malaria diagnosis by mitochondrial DNA-based PCR assay.** *Clinical Microbiolol Infect* 2011, **17:**1484-1491.

7. Tanizaki R, Ujiie M, Kato Y, Iwagami M, Hashimoto A, Kutsuna S, Hayakawa N, Kanagawa S, Kano S, Ohmagari N: **First case of Plasmodium knowlesi infection in a Japanese traveller returning from Malaysia.** *Malaria Journal* 2013, **12:**1-11.

8. Prugnolle F, Durand P, Neel C, Ollomo B, Ayala F, Arnathau C, Etienne L, Mpoudi-Ngole E, Nkoghe D, Leroy E, et al: **African great apes are natural hosts of multiple related malaria species, including Plasmodium falciparum.** *PNAS* 2010, **107:**1458-1463.
