# Supplementary material for: Non-invasive surveillance for Plasmodium in reservoir macaque species
Source: Malar J. 2015 Oct 12;14:404. doi: 10.1186/s12936-015-0857-2 (PMC4603874; doi:10.1186/s12936-015-0857-2)
Supplement: Additional file 3: — Optimal storage techniques. Data on DNA yield from faecal samples stored with four different protocols that varied in temperature and media. [file 12936_2015_857_MOESM3_ESM.docx]

**Additional file 3. Optimal storage techniques.**

In order to optimize collection and storage of fecal samples for downstream analysis of *Plasmodium* isolation and identification, we collected and stored fecal samples with four treatments. We examined DNA yield with two different storage media (RNAlater^©^ and 70% EtOH) and at two temperatures: room temperature (~20°C) and freezing (-20°C). Stoarge media was added in a 1:1 ratio. All RT samples were moved to freezers after two weeks, simulating field conditions. The treatments were as follows:

1. RNAlater^©^, room temperature (~20°C) for 2 weeks and then frozen until extraction
2. RNAlater^©^, immediately frozen (-20°C)
3. 70% ethanol, room temperature (~20°C) for 2 weeks and then frozen until extraction
4. 70% ethanol, immediately frozen (-20°C)

The samples came from four *Macaca nemestrina.* These individuals were in separate cages and blood samples were obtained for routine screening at the same time fecal samples were collected. Replicate samples from each individual were stored at each treatment, meaning there were eight samples per individual and thirty-two samples overall.

Each treatment group only has eight samples, thus the large variance in expected DNA yield. The variation between individuals was not greater than the variance among an individual. Even though DNA yield isn’t specific to parasite DNA, we have found that a high DNA yield is a useful predictor of success in amplifying parasite DNA.

RNAlater^©^ was the most consistent across temperatures, however there is no significant difference between Ethanol -20°C, RNAlater -20°C and RNAlater 20°C.

Figure S1. DNA Yield of fecal sample extractions (measured in a nanodrop). The mean of a treatment is given by the solid horizontal line, whereas the shaded area (blue or grey) gives the interquartile range (IQR). The dotted lines represent the minimum and maximum values, all of which were above room temperature ethanol.
